# Recovery of Vaginal Microbiota after Standard Treatment for Bacterial Vaginosis Infection: An Observational Study

**DOI:** 10.3390/microorganisms8060875

**Published:** 2020-06-09

**Authors:** Liisa Lehtoranta, Ashley A. Hibberd, Jenni Reimari, Jouni Junnila, Nicolas Yeung, Johanna Maukonen, Gordon Crawford, Arthur C. Ouwehand

**Affiliations:** 1 Global Health and Nutrition Science, DuPont Nutrition & Biosciences, Sokeritehtaantie 20, FIN-02460 Kantvik, Finland; jenni.reimari@dupont.com (J.R.); nicolas.yeung@dupont.com (N.Y.); pia-johanna.maukonen@dupont.com (J.M.); arthur.ouwehand@dupont.com (A.C.O.); 2 Genomics & Microbiome Science, DuPont Nutrition & Biosciences, 4300 Duncan Avenue, Saint Louis, MO 63110, USA; ashley.hibberd@dupont.com; 3 4Pharma Ltd., Arkadiankatu 7, 00100 Helsinki, Finland; jouni.junnila@4pharma.com; 4 CPS Research, 3 Todd Campus, West of Scotland Science Park, Glasgow G20 0SP, UK; gordon.crawford@cpsresearch.co.uk

**Keywords:** vaginal microbiota, bacterial vaginosis, *Lactobacillus*, Nugent score

## Abstract

Vaginal microbiota dysbiosis and bacterial vaginosis (BV) affect negatively women’s health. Understanding vaginal microbiota fluctuations in BV during and after antibiotic treatment would facilitate accurate decision-making on the treatment regimen, avoid unnecessary antibiotic use, and potentially mitigate recurrence. We investigated vaginal microbiota composition of 30 women with BV before and after 5-day metronidazole treatment and compared the results with 30 healthy women. Vaginal microbiota was assessed by Nugent score and analyzed by 16S rRNA gene sequencing in swabs on baseline Day 1, and on Day 8 and 15, after completion of antibiotic treatment by women with BV. Prior to antibiotic treatment (Day 1), BV-positive women were dominated by *Lactobacillus iners* (25.8%), *Prevotella timonensis/bivia* (18.0%), and *Gardnerella vaginalis* (14.6%), whereas healthy women were dominated by *L. iners* (37.5%) and *Lactobacillus crispatus/acidophilus* (19.2%). On Day 8, *L. iners* abundance increased in BV-treated women being significantly higher compared with healthy women (67.8% vs. 37.5%, *p* = 0.049). On Day 15, the relative abundance of all microbial taxa was similar between the groups. Vaginal microbiota of women with BV shifted to resemble that of healthy controls after metronidazole. Sequencing analysis provides more in-depth understanding of changes in vaginal microbiota. The role of *L. iners* in vaginal health and dysbiosis requires further investigations.

## 1. Introduction

Vaginal lactobacilli play a fundamental role in women’s health by interacting with host physiology and influencing pathogenic micro-organisms, thus positively affecting reproductive health and wellbeing. Understanding of vaginal microbiota communities and the impact on health has increased rapidly with the advent of molecular culture-independent methodologies. Vaginal microbiota cluster at least into five major community state types (CSTs). Four of them are dominated by lactobacilli, namely CST I by *Lactobacillus crispatus*, CST II by *Lactobacillus gasseri*, CST III by *Lactobacillus iners*, and CST V by *Lactobacillus jensenii* [1]. *L. crispatus, L. gasseri*, and *L. jensenii* are most strongly associated with healthy microbiota [1]. Thus far, *L. iners* is considered as the most prevalent species, both in healthy women and in women with vaginal dysbiosis and diseases [2]. CST IV is comprised of a mixture of strict and facultative anaerobes, including representatives of *Gardnerella*, *Atopobium*, *Sneathia*, and *Prevotella*. In fact, the bacterial composition of bacterial vaginosis (BV), one of the most common causes of vaginal discomfort in women, resembles CST IV in terms of lactobacilli depletion and the overgrowth of anaerobic bacteria such as *Gardnerella, Atopobium*, and *Prevotella*. BV is often accompanied with clinical symptoms such as irritation, abnormal vaginal discharge, and odor. However, some women with BV may be completely asymptomatic.

BV-associated bacteria e.g., *Gardnerella vaginalis* form biofilms [3], which may be one of the key reasons why up to 10% to 15% of BV patients fail to respond to initial antimicrobial therapy [4,5]. In addition, recurrence rates among responders remain significant [6], necessitating repeated administration of antibiotics. Repeated antibiotic exposure increases the risk of emergence of resistant strains, disruption of host-associated microbiota, and possible persistence of BV-associated microbial imbalance.

Clinical diagnosis of BV commonly relies on Nugent scoring accompanied with Amsel criteria. However, the diverse morphologies of vaginal bacteria, asymptomatic nature of BV, and subjectivity of microscopic examination complicates accurate and timely BV diagnosis. Especially *L. iners* with reported variable morphology may be overlooked by Gram straining [2]. Sequence-based applications provide deeper understanding of the vaginal microbiota community, and investigating dynamic changes in this community in BV during and after antibiotic therapy would be more informative in earlier evaluation and treatment of BV as well as predicting the recurrence risk [7]. Nevertheless, the information on the recovery period for when the microbiota returns to healthy state following BV treatment with antibiotic is limited [8]. This information would aid in making more accurate decisions in the treatment regimen. Therefore, the objective of this observational study is to investigate the time required both for the vaginal microbiota community and the Nugent score to return to normal after standard metronidazole treatment of BV infection by comparing the vaginal microbiota of BV-positive women with healthy women. In the microbiota sequencing analysis, we applied a denoising algorithm to account for errors in sequencing, which provided more resolution of the *Lactobacillus* species present in the vaginal microbiota. We found that vaginal microbiota of BV-positive women recovers to resemble that of healthy women after metronidazole cessation, but also discovered relatively high presence of *L. iners* in both healthy and women with BV at all time points.

## 2. Materials and Methods

### 2.1. Study Design

This was a non-interventional observational study conducted in Scotland, United Kingdom, from September to November 2017 (Clinicaltrials.gov identifier: NCT03187457). The study was approved on July 12th, 2017 by the East of Scotland Research Ethics Service, Regional Ethics Committee 2 (reference number 17/ES/0072). The study followed the ICH GCP guidelines and was conducted according to the ethical standards established in the 1964 Declaration of Helsinki. The sample size estimation was based on the judgement by the principal investigator and a study by Mayer et al. [8]. However, because of the nature of observational study design, no formal statistical analysis plan was prepared or power calculation was performed.

Written informed consent was obtained from all individual participants included into the study. The participants in Group 1 consisted of healthy women, asymptomatic for BV, whereas participants in Group 2 consisted of women at risk of BV infection, defined initially as ongoing self-evaluated BV symptoms (vaginal itching, vaginal discharge (thin and watery and either white or pale grey in color), irritation, burning, redness, swelling, pain or a rash). The symptoms were further reviewed and confirmed by the investigating physician. The inclusion criteria for all participants are as follows: signed informed consent, female gender, over 18 years of age, high probability for compliance with and completion of the study. Vaginal pH test Canestest (Bayer plc, Newbury, United Kingdom) was utilized as a screening tool for allocating participants into the study groups to evaluate the applicability of the test in future studies. For Group 1, the additional inclusion criterion was a negative Canestest result, whereas for Group 2 the Canestest result had to be positive. The participants were excluded if they were hypersensitive to metronidazole (only applied to Group 2), post-menopausal (defined as at least 12 consecutive months without menstruation), had received treatment for BV in last 4 weeks, had clinically significant menstrual irregularities, had suspected presence of sexually transmitted diseases (STDs) (such as *Chlamydia* or trichomoniasis) or other vaginal infections as judged by the investigating physician, were pregnant or breast feeding, used contraceptive methods containing spermicidal agents excluding condoms with spermicidal agents, participated in other clinical studies which could influence genitourinary tract microbiota, were unwilling to refrain from use of any other oral or vaginal probiotics during the study (only applied to Group 2), were abusing substances, the investigating physician suspected that participant is at high risk of STDs, or the investigating physician believed that the participant might be uncooperative and/or noncompliant and should therefore not participate in the study.

Healthy participants for Group 1 were recruited during routine care visits to their general practitioners. During baseline visit (referred to as Day 1), the current and history of vaginal infection (during the last three months) was obtained and a Canestest swab was collected. Participants with a negative Canestest underwent additional vaginal swabbing for Nugent scoring and for microbiota analysis which completed their study participation. Participants who had a positive Canestest were offered the opportunity to participate in Group 2 on the condition that also all other inclusion and exclusion criteria for Group 2 were applicable.

Participants for Group 2 were first screened online or by telephone. Those eligible (defined as ongoing self-evaluated BV symptoms or positive Canestest) were invited to baseline visit (referred to as Day 1) to the study clinic where the inclusion and exclusion criteria were further reviewed. During this visit, information on vaginal infections during the last three months and current vaginal symptoms were assessed and a Canestest swab was taken. Participants with a positive Canestest underwent vaginal swabbing for Nugent scoring and for microbiota analysis. Participants were then prescribed oral metronidazole; 400 mg, 3 times daily for 5 days. Participants were asked to refrain from sexual intercourse and use of intravaginal douches and creams for 24 h prior to the vaginal swab sampling at two follow-up visits: Day 8 (between 8–10 days from the baseline visit) and Day 15 (between 15–17 days from the baseline visit). At these follow-up visits, the symptoms and enquiry of compliance with metronidazole were performed and vaginal swabs were collected for Nugent scoring and for microbiota analysis.

### 2.2. Outcomes

The primary outcomes were to investigate the time required for the vaginal microbiota (particularly *Lactobacillus* abundance) and the Nugent score to return to normal after treatment of BV with metronidazole and compare the data to healthy women. Moreover, correlations between microbial taxa and the Nugent scores were analyzed as an exploratory outcome. Safety was evaluated by enquiring the participants on adverse events (AEs) at all visits. AEs were classified according to Medical Dictionary for Regulatory Activities (MedDRA).

### 2.3. Sample Collection

The vaginal samples were collected from the vaginal walls by gently rotating for 10–15 s by the study participants using polyester swabs (Becton Dickinson, Sparks, MD, USA). The swab was inserted in a collection tube (Vaginal Specimen Transport for the BD ProbeTec Qx Amplified DNA Assays; Becton Dickinson) carefully avoiding skin contamination. For Nugent score analysis, swab samples were transported from the research site to the Nuffield Hospital, Glasgow, United Kingdom. For the vaginal microbiota analysis, the swab samples as such were stored immediately at −20 °C after collection, and then transferred into −80 °C for longer term storage within 1 month of collection.

### 2.4. Nugent Score

The Nugent score was categorized as: no BV (0–3 score), intermediate (4–6 score), or BV (7–10 score) [9]. The differences between the study groups in absolute Nugent score values were analyzed with Wilcoxon rank-sum tests. The difference between days 8 and 15 within Group 2 was analyzed with Wilcoxon matched-pairs signed rank test.

### 2.5. Vaginal Microbiota Sample Processing and Analysis

#### 2.5.1. DNA Extraction

DNA from vaginal swabs was extracted using the Thermo Fisher Scientific (Waltham, MA, USA) MagMAX Express 96 and AM1840 Total Nucleic Acid Isolation Kit. Swab heads were clipped into 1.5 mL tubes and 550 µl of lysis binding concentrate was added. Then samples were shaken on an orbital dry bath at 900 rounds per minute (RPM) and 56 °C for 3 h. Subsequently, 400 µl of the sample was bead beaten (3 × 30 s at 6800 RPM) with the Precellys 24 homogenizer (Bertin Instruments, Montigny-le Bretonneux, France) and Precellys VK01 glass bead tubes. The tubes were then centrifuged at 16,000 RPM for 6 min and 115 µl of the supernatant was run on the MagMAX™ Pathogen RNA/DNA Kit, script 4462359_DW_HV (Thermo Fisher Scientific, Waltham, MA, USA).

#### 2.5.2. Vaginal Microbiota Sequencing and Data Analysis

The vaginal microbiota composition was analyzed by triplicate PCR amplification of the V4 variable region of the 16S rRNA gene using primers 515F (5′-GTGCCAGCMGCCGCGGTAA) and 806R (5′-GGACTACHVGGGTWTCTAAT) as previously described [10,11]. The amplicon library was sequenced using the Illumina MiSeq platform generating 2 × 250 base pair (bp) reads. Sequencing data were analyzed using QIIME2 (v. 2018.6) and QIIME1 (v. 1.9.1) [12]. One sample in the Group 1 was removed from the analyses because of insufficient sequence depth. Sequencing reads were demultiplexed using “qiime demux,” and “qiime dada2” was used to error-model and correct the Illumina reads, including denoising, dereplication, and removal of chimeras using the “consensus” method [13]. The reverse reads were truncated at 160 bp because of decreasing quality scores. Taxonomy was assigned to aligned amplicon sequence variants (ASVs; single-nucleotide difference sequences) using “q2-feature-classifier” (classify-sklearn) trained on the Greengenes version 13.8 database using 515F/806R 99% operational taxonomic units (OTUs) [14,15,16].

Species designation for ASVs from genera *Lactobacillus*, *Gardnerella*, and *Prevotella* were manually curated (noted by parenthesis in taxa labels) based on 100% similarity to type strain(s) in the EZ-Taxon database [17]. Taxa compositions summarized at species level are reported as relative abundance (% of total sequences), and were visualized using Prism (GraphPad Software, v. 7.0, La Jolla, CA, USA). Differentially abundant taxa (>0.1% abundance) were determined by Mann-Whitney U tests, and *p*-values were adjusted by Benjamini-Hochberg False Discovery Rate (FDR) in QIIME1 [18]. Average-linkage hierarchical clustering was conducted using pairwise Euclidean sample distance with log10-transformed species abundance in QIIME2 and assignment to previously defined CSTs [1].

Diversity comparisons were calculated using “qiime diversity core-metrics-phylogenetic” and “qiime group-significance” with 14,076 sequences per sample. The alpha diversity metric phylogenetic diversity (PD) whole tree [19] was compared using Kruskal-Wallis tests. Beta diversity (pairwise dissimilarity) was calculated using weighted UniFrac [20] and compared by permutational multivariate ANOVA (PERMANOVA). The distance matrix was visualized using principal coordinates analysis (PCoA) with the R (v. 3.4) ggplot2 package [21,22]. Spearman correlation analyses were conducted for microbial co-occurrence, α-diversity, and to Nugent scores with the R packages hmisc and gplots [23,24]. *p*-values were adjusted for multiple comparisons by FDR as noted. Sequencing data generated and/or analyzed during the current study are not publicly available and cannot be shared because of lack of consent from the study participants.

## 3. Results

### 3.1. Participant Flow

**Figure 1:**
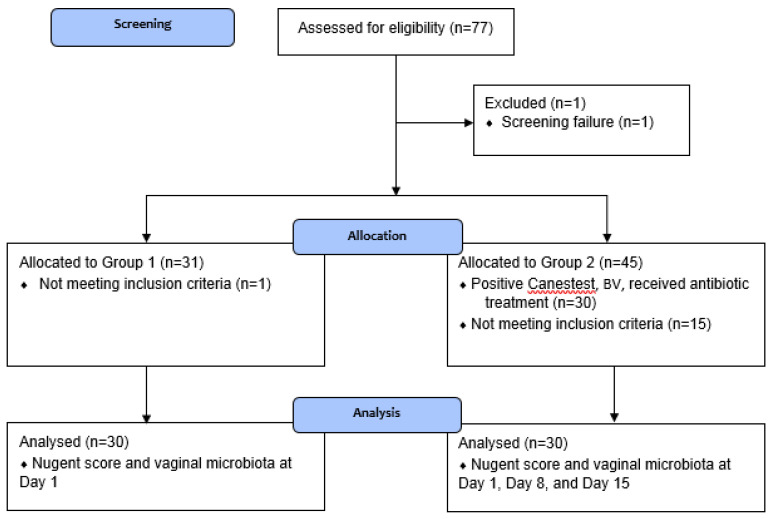
Flow of participants in the study.

**Figure 2:**
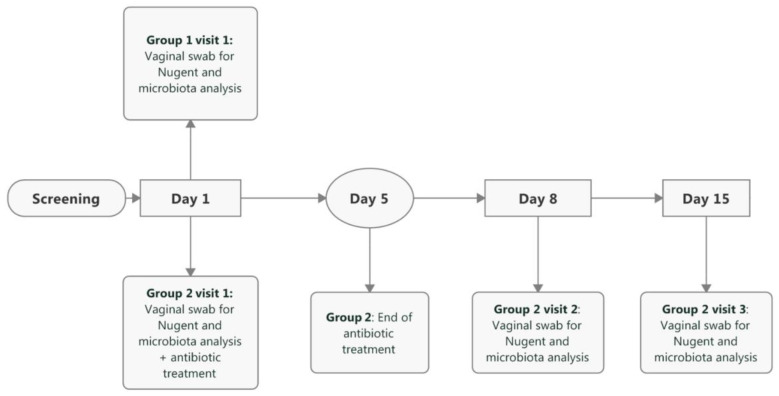
Timeline and schedule of events in the study. Baseline visit for both study groups is referred as Day 1. In Group 2, follow-up visits occurred between 8–10 days from the baseline visit (referred as Day 8) and between 15–17 days from the baseline visit (referred as Day 15).

Figure 1 shows the participant flow in the study and Figure 2 shows the study timeline. In total, 77 women (primarily Caucasian) were screened, of which one participant was a screen failure (Canestest-negative, but symptomatic for BV). In total, 30 healthy women (23–51 years; mean 35.4 years) were included in Group 1 and equally 30 BV-positive women (20–49 years, mean 32.6 years) in Group 2. One of the inclusion criteria for healthy women in Group 1 was a negative Canestest result on Day 1. In Group 1, 31 participants had negative Canestest result. However, one participant had BV symptoms and was excluded and offered to participate in Group 2. In Group 2 of BV infected women, one of the inclusion criteria was a positive Canestest result. Altogether, 45 participants were tested and 30 had positive Canestest result, whereas 15 tested negative and thus were excluded from the study. All 30 participants in Group 1 (1 visit) and Group 2 (3 visits) completed the study.

### 3.2. Nugent Score Evaluation

First, we investigated the time required for the Nugent score to return to normal (below 7) after treatment of BV with metronidazole. Healthy women in Group 1 served as reference for statistical comparisons. Table 1 shows the Nugent scores by study day in both study groups. In Group 1, most participants (21/30) had Nugent scores between 0–3. The Nugent scores in Group 2 on Day 1 prior to antibiotic treatment differed significantly from Group 1 (*p* = 0.001), as 50% of participants had a Nugent score ≥7, whereas the remaining 50% had either intermediate Nugent scores in between 4–6 (4/30) or 0–3 (11/30), despite the fact that these participants were all suffering from BV symptoms and had positive Canestest result, as judged by the clinician. After antibiotic treatment on Day 8, a clear decrease was observed in the Nugent scores, as 90% of the participants had a Nugent score below 7, and the majority (83%) with Nugent score below 4. On Day 8 (Group 2) vs. Day 1 (Group 1), Nugent scores were similar between the study groups. On Day 15 in Group 2, higher Nugent scores were recorded, and the scores were significantly different compared with Day 8 (*p* = 0.028). However, the Nugent scores on Day 15 in Group 2 were not different compared with Nugent scores on Day 1 in Group 1.

Table S1 shows individual Nugent scores for Group 2. In two participants with Nugent score ≥7 on Day 1, the Nugent scores remained ≥7 on Day 8. On Day 15, one participants Nugent score remained at 7. While, in one participant with Nugent score below 3 on Day 1, the Nugent score increased to 6 on Day 8 and to 8 on Day 15.

### 3.3. Vaginal Microbiota Evaluation

Second, from 16S rRNA gene sequencing data, we analyzed the vaginal microbiota diversity and the relative abundance of microbial taxa and compared the findings between study groups and time points. In addition, we analyzed the correlations of the microbial species to Nugent score and to each other.

#### 3.3.1. Diversity

**Figure 3:**
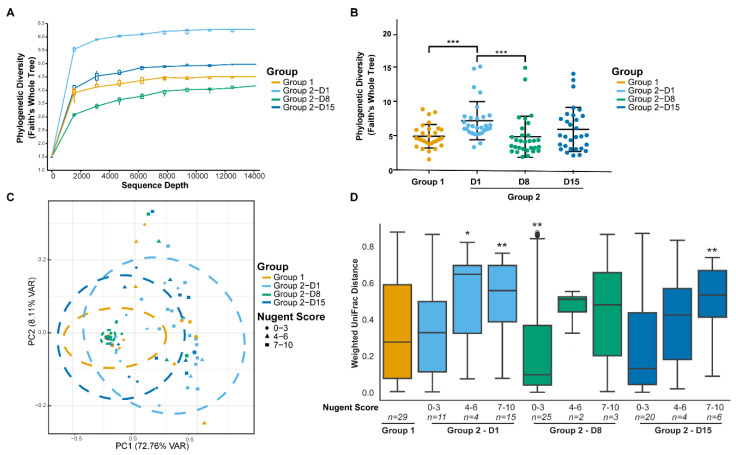
Alpha diversity of vaginal microbiota samples from individuals plotted as rarefaction curves (**A**) and compared between study groups and day using the phylogenetic diversity (Faith’s whole tree) metric (**B**). Beta diversity (weighted UniFrac metric) showing clustering of the vaginal microbiota communities from individuals by study group, day, and Nugent score in a principal coordinate analysis (PCoA) plot (**C**) and distances between Group 1 and Group 2 time points subgrouped by Nugent scores (**D**). Ellipses represent 95% confidence interval. ***FDR < 0.001, **FDR < 0.01, *FDR < 0.05; Kruskal-Wallis test.

As expected, there were differences in the microbial diversity between the study groups and time points prior to and after antibiotic treatment. α-diversity (within-sample species diversity) showed that rarefaction curves were saturated, indicating the sequencing depth was sufficient to capture the microbial diversity (Figure 3A). Faith’s PD whole tree metric (phylogenetic distance) (Figure 3B), was significantly lower in Group 1 when compared with Group 2 on Day 1. α-diversity also positively correlated with Nugent score (ρ = 0.5125, *p* < 0.001). Microbial diversity was significantly reduced after antibiotic treatment on Day 8. There were no statistically significant differences in the α -diversity between the study groups on Day 8 or Day 15. β-diversity (between-sample dissimilarity) based on the weighted UniFrac distance showed that clustering of the vaginal microbiota composition from individuals of Group 1 differed significantly from Group 2 on Day 1, prior to antibiotic treatment (PERMANOVA; FDR = 0.002) (Figure 3C). There was no statistical difference between the study groups on Day 8 or Day 15. For Group 2, β-diversity on Day 1 differed significantly from Day 8 and from Day 15, after antibiotic treatment (PERMANOVA; FDR = 0.002 for both). The highest similarity in β-diversity occurred between the study groups on Day 15.

#### 3.3.2. Relative Abundance

**Figure 4:**
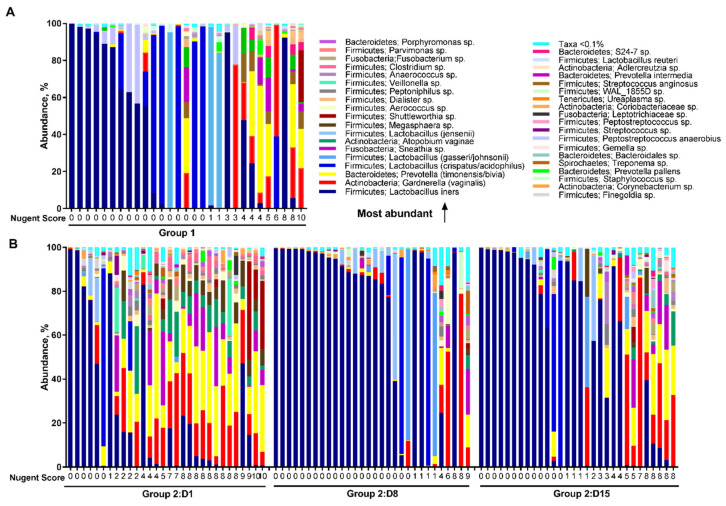
The relative abundance of vaginal microbiota species for participants in Group 1 (**A**) and Group 2 (**B**), in order of study day and increasing Nugent score.

There were apparent differences in the relative abundance of microbial species between the study groups and study days (Figure 4). Overall in healthy women in Group 1, the most abundant species in the vaginal microbiota were *L. iners* (37.5%) followed by *L. crispatus/acidophilus* (19.2%), *G. vaginalis* (9.6%), and *L. gasseri/johnsonii* (9.5%) (Figure 4A). In BV-positive women in Group 2 on Day 1, prior to antibiotic treatment, the most abundant species were *L. iners* (25.8%), *Prevotella timonensis/bivia* (18.0%), and *G. vaginalis* (14.6%). The presence of *L. iners* may explain the Nugent scores below 7 on Day 1 in Group 2 (Figure 4B). After treatment on Day 8, microbiota was dominated by the high abundance of *L. iners* (67.8%). Similarly, on Day 15, the most abundant species was *L. iners* (55.6%) followed by *G. vaginalis* (11.5%).

On Day 1, the vaginal microbiota composition differed significantly between the study groups (Figure 4, Table 2). In Group 1 there was a higher abundance in *L. crispatus/acidophilus* (19.2% vs. 3.5%, FDR = 0.042), and *L. jensenii* (4.6% vs. 1.3%, FDR = 0.018) compared with Group 2. In contrast in Group 2, there was a higher abundance of members of *P. timonensis/bivia* (18.0% vs. 7.6%, FDR = 0.003), *G. vaginalis* (14.6% vs. 9.6%, FDR = 0.018), and *Atopobium vaginae* (5.9% vs. 1.8%, FDR = 0.018) when compared with Group 1. In addition, significantly higher abundance was observed in Group 2 over Group 1 with members of genera *Peptoniphilus, Aerococcus, Dialister, Parvimonas, Clostridium, Porphyromonas, Sneathia, Adlercreutzia, Bacteroidales, Megasphaera*, and *Anaerococcus* (FDR < 0.05).

On Day 8, after antibiotic treatment, there were less differences in the relative abundance of microbial species between the study groups than prior to antibiotic treatment. The abundance of *L. iners* was significantly higher in Group 2 (67.8% vs. 37.5%, FDR = 0.049) when compared with Group 1. In contrast, *P. timonensis/bivia* was significantly lower in Group 2 (1.4% vs. 7.6%, FDR = 0.049) when compared with Group 1. Similarly, members of *Finegoldia, Anaerococcus*, and *Peptoniphilus* were significantly lower in Group 2 (FDR < 0.05). When the time points within Group 2 were compared, *L. iners* significantly increased from 25.8% on Day 1 to 67.8% on Day 8 (FDR = 0.003) following the antibiotic treatment. In contrast, the abundance of most non-lactobacilli species significantly decreased from Day 1 to Day 8, and then increased moderately by Day 15.

On Day 15, there were no significant differences in the relative abundance of microbial taxa either at genus or species level between the study groups (FDR > 0.05), indicating that the vaginal microbiota shifted to resemble the microbiota of healthy reference women i.e., 10–12 days after completion of the antibiotic treatment.

#### 3.3.3. Nugent Score Subgroups within Group 2

**Figure 5:**
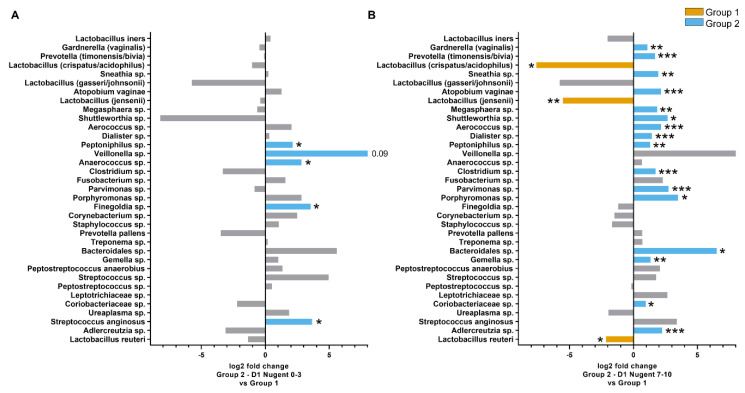
Differences in the relative abundance of species in the vaginal microbiota between Group 1 and Group 2 Day 1 individuals with Nugent scores 0–3 (**A**) Group 2 Day 1 individuals with Nugent scores 7–10 (**B**). Taxa are listed in order of overall total abundance. ***FDR < 0.001, **FDR < 0.01, *FDR < 0.05.

An additional analysis was conducted to investigate differences in the microbiota composition where the Group 2 time points were further subgrouped according to Nugent scores (0–3, 4–6, and 7–10). The weighted UniFrac distances within Group 1 and the Group 2 Nugent score subgroups are shown in Figure 3D. The within-group distances were lowest for Group 2 with Nugent scores 0–3 at both Day 8 and Day 15, indicating the microbiota composition of samples within these subgroups were highly similar to each other. On Day 1, the UniFrac distances between Group 1 and Group 2 subgroups were significantly different for Nugent scores 4–6 and 7–10 (FDR = 0.038 and 0.005, respectively) but not for Nugent scores 0–3 (FDR > 0.1). The majority of individuals on Day 8 had Nugent scores 0–3 and differed significantly from Group 1 (FDR = 0.009). By Day 15, Group 2 individuals did not differ significantly from Group 1 (FDR > 0.1), except for six individuals that had Nugent scores 7–10 (FDR = 0.007). Although the overall microbiota composition for Group 2 individuals with Nugent scores 0–3 on Day 1 did not differ from Group 1 according to the UniFrac distance, the relative abundance of some individual species differed between the groups. The abundance of *Peptoniphilus* sp. (FDR = 0.036), *Anaerococcus* sp. (FDR = 0.036), *Finegoldia* sp. (FDR = 0.020), and *Streptococcus anginosis* (FDR = 0.05) was four times greater in Group 2 individuals with Nugent scores 0–3 compared to Group 1 (Figure 5A). Of these species, only *Peptoniphilus* sp. was more abundant in Group 2 individuals with Nugent scores 7–10 compared to Group 1 (Figure 5B). Other species that were enriched in Group 2 individuals with Nugent scores 7–10 compared to Group 1 included *G. vaginalis*, *P. timonensis/bivia*, *Sneathia* sp., *A. vaginae*, *Megasphaera* sp., and several minor species, while *L. crispatus/acidophilus*, *L. jensenii*, and *L. reuteri* were reduced (FDR < 0.05; Figure 5B).

#### 3.3.4. Community State Types

**Figure 6:**
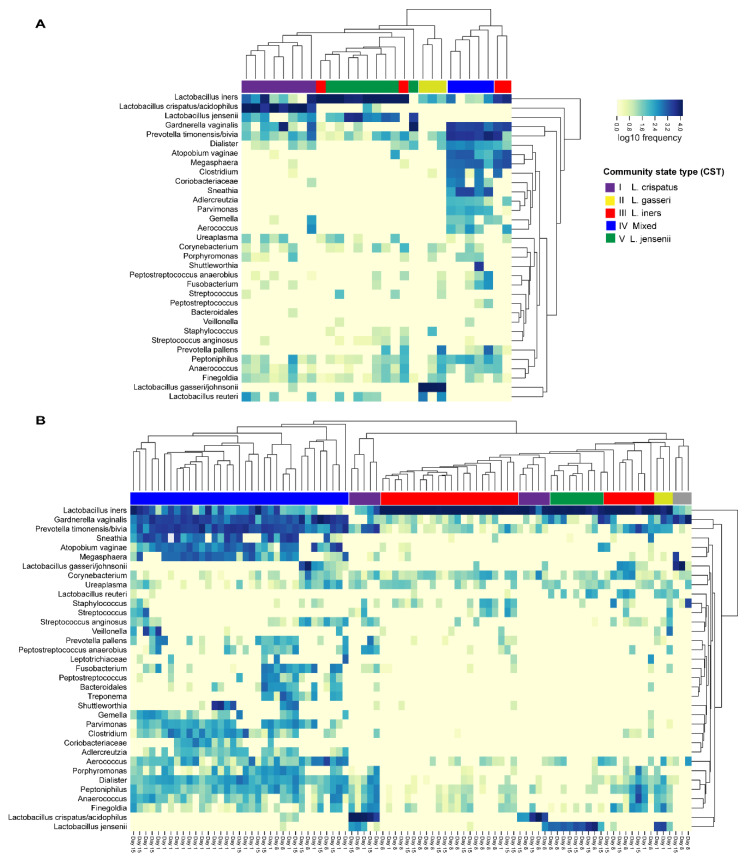
Distribution of vaginal microbiota community state types (CSTs) within (**A**) Group 1 (30 healthy women without BV symptoms, vaginal pH < 4.5) and within (**B**) Group 2 (30 BV- positive women, vaginal pH > 4.5) on Day 1, Day 8, and Day 15 by hierarchical clustering.

The vaginal microbiota composition was also grouped into CSTs by hierarchical clustering and colored according to the predominant species (Figure 6). Figure 6 shows the clustering in both study groups separately and Figure S1 shows the clustering when the study groups are combined. Notably in both study groups, *L. iners* was present in all CST clusters with relatively high abundance, and CST III (*L. iners* dominated) was represented by more than one cluster. The vaginal microbiota in Group 1 was mostly represented with CST I (*L. crispatus*) and CST V (*L. jensenii*) (Figure 6A). In contrast, the vaginal microbiota in Group 2 on Day 1 were mostly represented with CST IV (mixed) or CST III (*L. iners*) (Figure 6B). After antibiotic treatment on Day 8 and Day 15, the communities were markedly changed with increased dominance of CST III (*L. iners*). On Day 15, there was an increase with CST IV (mixed) when compared with Day 1 and Day 8. When the study groups were combined into the clustering analysis (Figure S1) the majority of samples in Group 1 clustered together with Group 2 Day 8 and/or Day 15 samples with the exception of CST I (*L. crispatus*-dominated), which was largely represented by only Group 1 samples.

#### 3.3.5. Correlations to Nugent Score and Taxa

**Figure 7:**
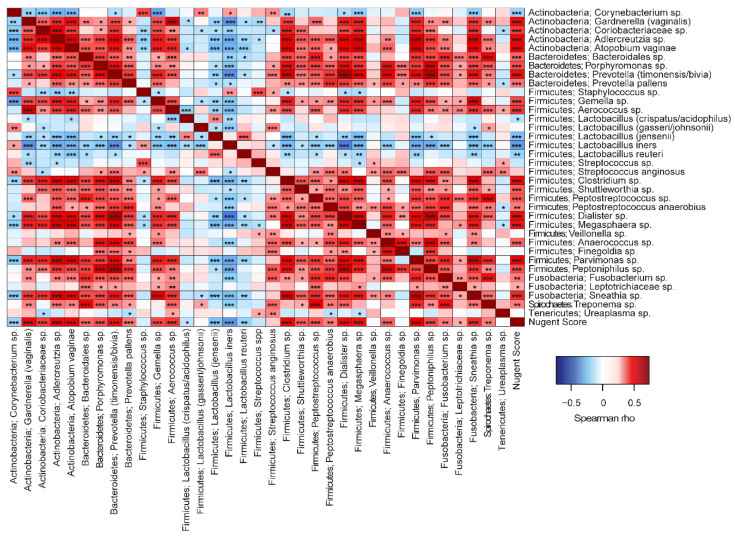
Spearman correlation analyses between vaginal microbiota species and to Nugent Score. ***p < 0.001, **p < 0.01, *p < 0.05.

**Figure 8:**
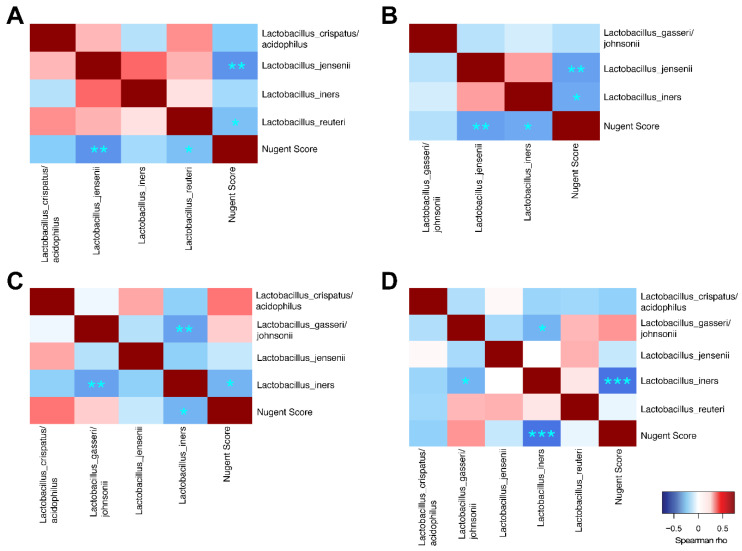
Spearman correlation analyses between vaginal *Lactobacillus* species and to Nugent score for Group 1 (**A**), Group 2 Day 1 (**B**), Group 2 Day 8 (**C**) and Group 2 Day 15 (**D**). ***p < 0.001, **p < 0.01, *p < 0.05.

Overall significant correlations were found between Nugent score and microbial taxa (Figure 7). Most taxa correlated positively with Nugent score, except for *Lactobacillus* spp. and *Corynebacterium* sp. In addition, there were significant positive correlations between the many of the non-lactobacilli species, indicating that these taxa favor co-occurrence. Conversely, *Lactobacillus* spp. generally correlated negatively to non-lactobacilli such as *G. vaginalis*, *Prevotella* spp., and *A. vaginae.* Overall, *L. jensenii* correlated positively with *L. crispatus/acidophilus* and with *L. reuteri*, but negatively with *L. gasseri. L. iners* also correlated negatively with *L. gasseri.* In Group 1, *L. jensenii* and *L. reuteri* correlated negatively with Nugent score (Figure 8A). In Group 2, *L. jensenii* showed negative correlation to Nugent score on Day 1 (Figure 8B). *L. iners* correlated negatively to Nugent score in Group 2 at all time points, but most strongly on Day 15 (Figure 8B–D). *L. gasseri* correlated negatively to *L. iners* on Day 8 and Day 15 (Figure 8C–D).

### 3.4. Safety

There were three AEs in the study: diarrhea, headache, and vomiting. None of the reported AEs were considered as treatment related or as serious.

## 4. Discussion

The present observational trial was designed to investigate in BV-infected women the recovery of vaginal microbiota community after oral metronidazole treatment and compare data with healthy reference women. Although information on recovery time would be valuable for clinicians when making decisions on the treatment regimen, only limited number of studies exist on this topic [8]. In the current study, the vaginal microbiota showed recovery within 3–5 days after treatment cessation, but recovery time varied depending on the analysis methodology (Nugent score vs. 16S RNA gene sequencing). Prior to treatment, the vaginal microbiota of women with BV was dominated by *L. iners*, *Prevotella timonensis/bivia*, and *G. vaginalis.* Three to five days after metronidazole treatment, the microbial community converted into high dominance of *L. iners.* These findings are in agreement with other longitudinal analyses studying changes in the BV-infected vaginal microbiota following antibiotic treatment [4,8,25,26]. Mayer et al. reported that the use of topical or oral metronidazole resulted in rapid clearance of BV-associated bacteria within a few days as analyzed by qPCR, but they also reported reemergence of BV-associated species such as *G. vaginalis* and *A. vaginae* following treatment cessation [8]. In the present study, Nugent scores between the study groups were similar on Day 8 (3–5 days) after cessation of metronidazole therapy. In contrast, based on the sequencing data, the relative abundance of bacterial species was significantly different at that time point between these women, and the similarity between the study groups was observed on Day 15 (10–12 days) when the antibiotic treatment was completed. Similarly, as in the study by Mayer et al. [8] we also noticed that the relative abundance of *G. vaginalis* and *P. timonensis/bivia* increased on Day 15 in some antibiotic-treated women. Potentially, these women were at subsequent risk for either relapse or reinfection with BV.

Nugent score is the gold standard in clinical use, whereas molecular methods, such as high throughput sequencing are more utilized in research settings. With Nugent scoring the interpretation of the bacterial staining is subjective, and the result can be biased if the abundance of bacteria in the glass smear is low. The obvious advantage with sequencing is that the method provides more in-depth data of the species present in the vaginal microbiota [27]. For the current dataset, we applied a denoising algorithm to the microbial sequence data analysis to account for sequencing error [13]. This algorithm defines sequence variants with single nucleotide differences rather than clustering sequences to OTUs, thus enabling more precise resolution of vaginal microbiota composition (especially lactobacilli) and BV-associated alterations before and after antibiotic therapy. For instance, sequencing analysis enabled us to identify the relatively high presence of *L. iners* at baseline in symptomatic BV-positive women, who had Nugent scores 0–3. Although BV diagnosis based on Nugent score for these women is contradictory, sequencing analysis showed that these women also had significantly higher abundance of *S. anginosus* and Gram-positive anaerobic cocci (*Peptoniphilus* sp., *Anaerococcus* sp., and *Finegoldia* sp.), which have been associated with BV/vaginal infections [28,29,30]. Given the non-specificity and subjectivity of Nugent scoring method, our study results are aligned with previous recommendations that prospective BV treatment studies should be analyzed based on the microbiota and not exclusively on the Nugent score [27,31].

In our dataset, the most frequently abundant *Lactobacillus* species was *L. iners*, both among healthy women and among BV-positive women; at all time points. Our results are well aligned with previous studies, where *L. iners* has been detected in high levels both in BV-diagnosed and in healthy Caucasian women [2,32,33,34]. Therefore, the role of *L. iners* in vaginal health is very controversial. Suggestions exist that *L. iners* could be beneficial in supporting the recovery to a lactobacilli-dominated microbiota after dysbiosis, e.g., because of antibiotic treatment [4,35]. In our study cohort, the dominance of *L. iners* was apparent after 5-day metronidazole treatment, where the relative abundance of *L. iners* increased from 22% to 60% and 55% on days 8 and 15, respectively. However, as the study follow-up ended after study day 15, the subsequent risk for these women to further develop BV could not be evaluated. Nevertheless, data indicates that women colonized with *L. iners* appear to have a significantly higher risk for developing BV relative to women colonized with e.g., *L. crispatus* [36]. This may be due to the fact that unlike *L. crispatus, L. iners* is unable to synthesize D-lactic acid, which is more protective against vaginal dysbiosis than L-lactic acid [37,38]. In the current study; although the healthy reference women also had relatively high abundance of *L. iners*, it is important to note that they also had significantly higher abundance of *L. crispatus/acidophilus* when compared with the BV-infected women at baseline. Moreover, clustering analysis showed that the vaginal microbiota of the healthy women was dominated by *L. crispatus* (CST I) and *L. jensenii* (CST V). In addition, they had no symptoms for BV/other vaginal infections and had no history of vaginal infections from the last 3 months as judged by the physician. Interestingly, 3 of the 30 healthy women had Nugent score above 7. The vaginal microbiota of these women most probably resembled “molecular-BV” characterized by depletion of *Lactobacillus* spp. (CST IV) and reflect similar low-*Lactobacillus* states that are captured by Nugent score [39]. Nevertheless, as the study did not aim to explore the potential association of *Lactobacillus* species and BV risk, the role of *L. iners* in the pathogenicity of BV remains to be determined in future studies.

Based on the Nugent score, the response rate (decrease below 7) to metronidazole on Day 8 was 90% in our study cohort. Within the remaining non-responding 10%, two women had Nugent scores below 7 later on Day 15. One participant failed to respond to the antibiotic treatment. Our findings are in accordance with previous data showing limited efficacy of oral metronidazole [5,8,25]. However, we cannot rule out the possibility of misdiagnosis of BV by Nugent scoring in some cases instead of ineffective antibiotic treatment. For instance, in one of our non-responding participants with Nugent score 8 (unchanged from baseline) on Day 8, the sequencing analysis showed that the relative abundance of *L. iners* was highly increased compared with baseline (Figure 2 and Figure 5). It should be noted, however, that the interpretation of our results is potentially affected by the high abundance of *L. iners* and discrepancy between the Nugent scores. For example, although *L. iners* dominated most vaginal samples with Nugent score 0–3 and showed significant negative correlation, the species also dominated some samples with Nugent score ≥7. Interestingly, the morphology of *L. iners* may vary between isolates [2] highlighting the fact that microscopy-based diagnostic methods can bias the information on the vaginal microbiota composition and more research is needed to understand the impact of *L. iners* on Nugent scores and BV diagnosis.

We also applied correlation analysis to explore microbial interactions within the vaginal microbiota community. As expected, we found that *Lactobacillus* spp. correlated negatively to non-lactobacilli such as *G. vaginalis*, *Prevotella* spp., and *A. vaginae*. Interestingly, among *Lactobacillus* spp., *L. jensenii* correlated positively with *L. crispatus/acidophilus* and with *L. reuteri*, indicating that these taxa favor the co-occurrence and synergy. In contrast, *L. jensenii* and *L. iners* correlated negatively with *L. gasseri* indicating that these strains may have antagonistic effects to each other. Similar correlation has been reported by De Backer and colleagues [40]. Identification of these microbial interactions could aid in designing e.g., probiotic applications for maintaining healthy vaginal microbiota or help in restoring the microbiota after antibiotic treatment and reducing the recurrence rate.

In the present study, we used vaginal pH test (Canestest) as an additional screening tool when allocating participants into the study groups in order to evaluate the applicability of the pH test for screening in future pivotal studies. In the literature, self-testing for abnormal vaginal pH has been reported as a feasible preliminary screening tool to detect high risk for abnormal vaginal microbiota and BV [41]. The sensitivity of Canestest in our study cohort varied between the study groups as 10% of Canestest-negative healthy women (without BV symptoms) had Nugent score above 7, and 50% of Canestest-positive women with BV had Nugent score below 7. For future studies, Canestest could be a simple and cost-effective tool for initial screening but should not be solely used to discriminate BV and healthy women.

There are limitations in our observational study design that need to be addressed. The sample size is relatively small and the follow-up for BV-treated women ended on Day 15 (after 15–17 days). Therefore, the subsequent risk for recurrent BV for these women by characterizing their microbiota could not be calculated. In addition, the healthy women were sampled at only one time point. Furthermore, inclusion of Amsel criteria would have been valuable for confirmation of the BV diagnosis and provide more information on the role of *L. iners* in the dataset. Notably, the study population represent primarily Caucasian women, of whom the vaginal microbiota is different compared to e.g., women originating from Africa. Therefore, the results may not be applicable to other ethnic groups [42,43].

In summary, our results suggest that the vaginal microbiota of women treated for BV recovers to resemble the microbiota similar to a healthy one after completion of oral metronidazole therapy. Inclusion of microbiota sequencing analysis on top of Nugent score analysis would provide more comprehensive and accurate data for BV diagnosis, in particular when *L. iners* is the dominant species, and assist in timing of targeting potential novel therapies and prevention protocols for BV. Because of high abundance of *L. iners* in both healthy and women diagnosed with BV, its role in health and disease should be further explored in future studies.

## Figures and Tables

**Table 1:** Distribution of Nugent scores between the study groups and study visits.

Nugent Score	Group 1 Canestest Negative	Group 2 Canestest Positive		
	Day 1	Day 1	Day 8	Day 15
0–3	21	11	25	20
4–6	6	4	2	4
>7	3	15	3	6
**Total**	30	30	30	30

**Table 2:** Differences in the relative abundance of species in the vaginal microbiota between study groups and within Group 2 (BV-positive women).

	Relative Abundance (%), Mean ± SD				*p*-Value (FDR *adj.*)				
Taxon	Group 1	Group 2							
	*n* = 29	Day 1 *n* = 30	Day 8 *n* = 30	Day 15 *n* = 30	Group 1 vs. Group 2 Day 1	Group 1 vs. Group 2 Day 8	Group 2 Day 1 vs. Day 8	Group 2 Day 8 vs. Day 15	Group 2 Day 1 vs. Day 15
Actinobacteria									
*Adlercreutzia* sp.	0.1 ± 0.2	0.3 ± 0.4	0.01 ± 0.03	0.1 ± 0.2	0.018		<0.001		0.008
*Atopobium vaginae*	1.8 ± 3.9	5.9 ± 7.4	0.2 ± 1.2	1.2 ± 3.0	0.018		<0.001		0.011
*Coriobacteriaceae* sp.	0.5 ± 1.4	0.5 ± 1.2	0.00 ± 0.00	0.01 ± 0.01			0.001		0.008
*Corynebacterium* sp.	0.1 ± 0.1	0.2 ± 0.7	0.7 ± 1.4	0.1 ± 0.1		0.001	<0.001		
*Gardnerella vaginalis*	9.6 ± 18.6	14.6 ± 11.7	6.1 ± 16.9	11.5 ± 20.0	0.018		0.002		
Bacteroidetes									
*Bacteroidales* sp.	0.002 ± 0.01	0.2 ± 0.6	0.2 ± 0.6	0.3 ± 1.5	0.018				
*Porphyromonas* sp.	0.1 ± 0.1	0.6 ± 0.9	0.3 ± 1.0	0.4 ± 1.0	0.018		0.001	0.035	
*Prevotella pallens*	0.5 ± 1.7	0.5 ± 1.3	0.1 ± 0.3	0.3 ± 1.0			0.008	0.035	
*Prevotella (timonensis/bivia)*	7.6 ± 15.0	18.0 ± 14.9	1.4 ± 4.0	6.7 ± 9.9	0.003	0.049	<0.001		0.007
Firmicutes									
*Aerococcus* sp.	0.3 ± 0.8	1.1 ± 1.9	1.0 ± 3.8	0.4 ± 1.0	0.003				
*Anaerococcus* sp.	0.2 ± 0.3	0.6 ± 1.0	0.02 ± 0.1	1.2 ± 3.5	0.025	0.001	<0.001	<0.001	
*Clostridium* sp.	0.8 ± 2.0	1.6 ± 2.5	0.03 ± 0.1	0.1 ± 0.3	0.013		<0.001		0.008
*Dialister* sp.	0.6 ± 1.2	1.2 ± 1.0	0.1 ± 0.4	0.8 ± 1.6	0.004		<0.001		0.011
*Finegoldia* sp.	0.1 ± 0.2	0.6 ± 1.2	0.1 ± 0.5	0.5 ± 0.8		<0.001	<0.001	<0.001	
*Gemella* sp.	0.1 ± 0.3	0.3 ± 0.6	0.01 ± 0.5	0.4 ± 1.1			0.002		
*Lactobacillus (crispatus/acidophilus)*	19.2 ± 36.0	3.5 ± 15.2	6.8 ± 23.4	6.5 ± 20.9	0.042				
*Lactobacillus iners*	37.5 ± 41.3	25.8 ± 34.2	67.8 ± 41.0	55.6 ± 41.5		0.049	0.003		
*Lactobacillus (jensenii)*	4.6 ± 10.6	1.3 ± 4.8	3.4 ± 10.7	2.2 ± 7.6	0.018				
*Megasphaera* sp.	2.0 ± 4.2	4.5 ± 4.8	0.1 ± 0.6	1.3 ± 4.4	0.018		<0.001		0.008
*Parvimonas* sp.	0.2 ± 0.4	0.9 ± 1.0	0.1 ± 0.3	0.3 ± 0.9	0.004	<0.001	<0.001		0.009
*Peptoniphilus* sp.	0.3 ± 0.5	1.0 ± 1.1	0.2 ± 0.8	0.8 ± 1.8	0.003	0.049	<0.001	0.024	0.016
*Peptostreptococcus anaerobius*	0.1 ± 0.5	0.4 ± 0.8	0.02 ± 0.1	0.2 ± 0.7			<0.001		
Fusobacteria									
*Fusobacterium* sp.	0.1 ± 0.5	0.6 ± 1.5	0.2 ± 0.8	0.4 ± 1.3			0.046		
*Sneathia* sp.	2.0 ± 5.3	6.8 ± 10.3	1.2 ± 4.2	2.5 ± 6.3	0.018		0.011		

Mann-Whitney U test with Benjamini-Hochberg false discovery rate (FDR) adjustment. Only taxa that differed between time points with an FDR < 0.05 are shown.

## Supplementary Materials

The following are available online at https://www.mdpi.com/2076-2607/8/6/875/s1, Table S1: Nugent scores of participants in the Group 2 on Days 1 (baseline), 8, and 15. Figure S1. Distribution of vaginal microbiota community state types (CSTs) in combined study groups by hierarchical clustering (Group 1: healthy women; Group 2; women diagnosed with BV).
