# Supplementary material for: Recovery of Vaginal Microbiota after Standard Treatment for Bacterial Vaginosis Infection: An Observational Study
Source: Microorganisms. 2020 Jun 9;8(6):875. doi: 10.3390/microorganisms8060875 (PMC7355544; doi:10.3390/microorganisms8060875)
Supplement: Supplementary file 1 [file microorganisms-08-00875-s001.zip › Table S1.docx]

**Table S1.** Nugent scores of participants in the Group 2 on Days 1 (baseline), 8, and 15.

| **Subject number** | **Day 1** | **Day 8** | **Day 15** |
| --- | --- | --- | --- |
| 001-038 | 10 | 00 | 00 |
| 001-041 | 10 | 00 | 00 |
| 001-053 | 00 | 00 | 00 |
| 001-047 | 02 | 00 | 00 |
| 001-039 | 04 | 00 | 00 |
| 001-044 | 07 | 00 | 00 |
| 001-029 | 08 | 00 | 00 |
| 001-030 | 08 | 00 | 00 |
| 001-054 | 08 | 00 | 00 |
| 001-031 | 09 | 00 | 00 |
| 001-040 | 00 | 01 | 00 |
| 001-016 | 02 | 01 | 00 |
| 001-043 | 08 | 08 | 00 |
| 001-006 | 00 | 00 | 01 |
| 001-055 | 00 | 00 | 01 |
| 001-058 | 08 | 00 | 01 |
| 001-059 | 09 | 01 | 01 |
| 001-037 | 00 | 00 | 02 |
| 001-035 | 01 | 00 | 03 |
| 001-022 | 02 | 00 | 03 |
| 001-033 | 00 | 00 | 04 |
| 001-056 | 08 | 00 | 04 |
| 001-060 | 07 | 01 | 05 |
| 001-017 | 05 | 09 | 05 |
| 001-010 | 08 | 08 | 07 |
| 001-009 | 04 | 00 | 08 |
| 001-042 | 08 | 00 | 08 |
| 001-069 | 08 | 00 | 08 |
| 001-036 | 04 | 04 | 08 |
| 001-063 | 02 | 06 | 08 |
