# Supplementary figures and images for: Recovery of Vaginal Microbiota after Standard Treatment for Bacterial Vaginosis Infection: An Observational Study
Source: Microorganisms. 2020 Jun 9;8(6):875. doi: 10.3390/microorganisms8060875 (PMC7355544; doi:10.3390/microorganisms8060875)

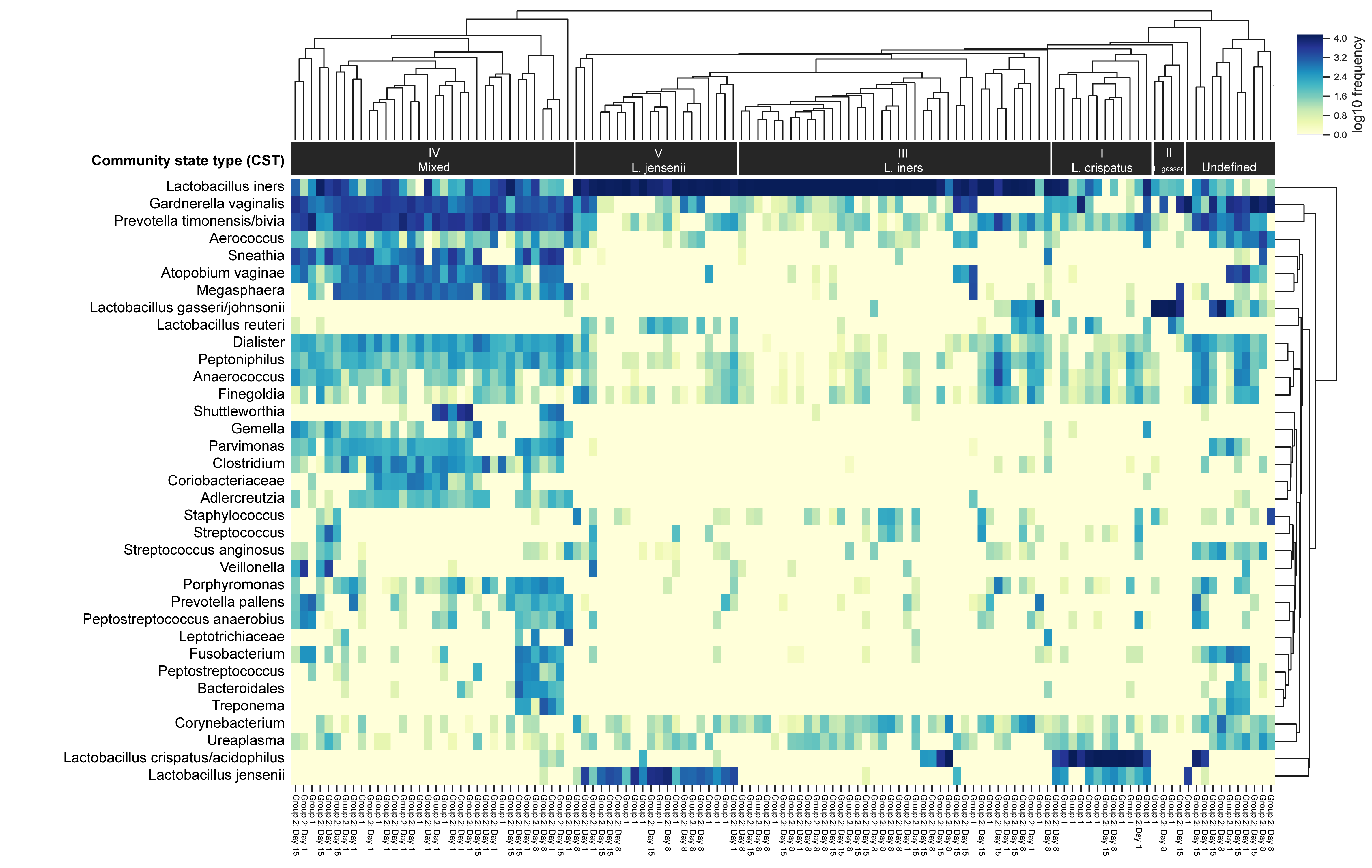

Supplement: Supplementary file 1 [file microorganisms-08-00875-s001.zip › Figure S1.tif]
